# Supplementary material for: Gene-SGAN: discovering disease subtypes with imaging and genetic signatures via multi-view weakly-supervised deep clustering
Source: Nat Commun. 2024 Jan 8;15:354. doi: 10.1038/s41467-023-44271-2 (PMC10774282; doi:10.1038/s41467-023-44271-2)
Supplement: Supplementary file 1 — Supplementary Information [file 41467_2023_44271_MOESM1_ESM.pdf]

Gene-SGAN: discovering disease subtypes with imaging and genetic signatures via multi-view weakly-supervised deep clustering

Supplementary Information

Supplementary Method 1: Gene-SGAN: supplementary methodological details.

Supplementary Method 1.1: Application of the inverse function for the subtype derivations.

Supplementary Method 1.2: Modification of the GAN loss in the Gene-SGAN model: Motivation and Derivation.

Supplementary Method 1.3: Derivation of the ELBO for the Gene Step.

Supplementary Method 2: Implementation details of the Gene-SGAN model.

Supplementary Method 2.1: Model Architectures.

Supplementary Method 2.2: Training Details.

Supplementary Method 3: Implementation details of model comparisons in the semi-synthetic experiments.

Supplementary Method 4: Replication analyses for SNP-subtype associations.

Supplementary Method 5: Adjusted N-Asso-SNPs for selecting the optimal number of clusters.

Supplementary Method 6: Examination of genetic and phenotypic variations captured by z_2 and z_3.

Supplementary Method 6.1: Associations of z_2 and z_3 with imaging volumetric measures.

Supplementary Method 6.2: Associations of z_2 and z_3 with AD-associated SNPs.

Supplementary Method 7: Reproducibility analyses of subtype memberships and latent variables.

Supplementary Method 7.1: Reproducibility analyses of AD-related subtypes.

Supplementary Method 7.2: Reproducibility analyses of hypertension-related subtypes.

Supplementary Note 1: Gene-SGAN produces reproducible subtypes and latent variables.

Supplementary Note 1.1: Gene-SGAN produces reproducible hypertension-related subtypes and latent variables.

Supplementary Note 1.2: Gene-SGAN produces reproducible AD-related subtypes and latent variables.

Supplementary Note 1.3: Gene-SGAN produces consistent subtypes and latent variables across different scales.

Supplementary Table 1: Network architectures of the function f.

Supplementary Table 2: Network architectures of the functions D, g_1&g_2, h, r.

Supplementary Table 3: Implementation details of six compared methods.

Supplementary Table 4: Reproducibility of AD-related subtypes in nested cross-validation.

Supplementary Table 5: Reproducibility of hypertension-related subtypes in stratified split-sampled experiments.
Supplementary Table 6: Reproducibiligy of hypertension-related subtypes in nested cross-validation.
Supplementary Table 7: Consistency of AD-related subtypes and latent variables across different model scales M.
Supplementary Table 8: Consistency of hypertension-related subtypes and latent variables across different model scales M.

Supplementary Algorithm 1: Gene-SGAN training procedure.

Supplementary Figure 1: Gene-SGAN identifies the simulated ground truth in semi-synthetic experiments.
Supplementary Figure 2: Gene-SGAN provides consistent and refined multiscale imaging subtypes related to AD.
Supplementary Figure 3: Gene-SGAN provides consistent and refined multiscale imaging subtypes related to hypertension.
Supplementary Figure 4: z_2 and z_3 variables capture additional non-linked imaging-specific and genetic-specific variations among MCI/AD participants.
Supplementary Figure 5: Gene-SGAN identifies hypertension-related subtypes with replicable imaging signatures in discovery and replication sets.

Supplementary Method 1: Gene-SGAN: supplementary methodological details

Supplementary Method 1.1: Application of the inverse functions for the subtype derivations.

The clustering function, g_1, is applied to the training and independent test data to derive their subtypes of interest. As introduced in **Method 1**, through GAN and regularization, we encourage that the transformation function approximates the underlying pathological process h. After the training process, we assume that $p_{syn}(f(\mathbf{x}, \mathbf{z_1}, \mathbf{z_2})) \approx p_{tar}(\mathbf{y})$ and that the function f satisfies the constraints imposed by regularization. Thus, we consider the learned transformation function f a good approximation of the underlying function h, such that $f(\mathbf{x}, \mathbf{z_1}, \mathbf{z_2}) \approx h(\mathbf{x}, \sigma_1(\mathbf{z_1}), \sigma_2(\mathbf{z_2}))$, where $\sigma_i \in \Omega_i$ and Ω_i is a class of permutation functions that change the order of elements in the latent variables $\mathbf{z_1}$ and $\mathbf{z_2}$. Since the orders of elements in the latent variables are unimportant and we can always change the orders of the derived subtypes, we rewrite the equation as $f(\mathbf{x}, \mathbf{z_1}, \mathbf{z_2}) \approx h(\mathbf{x}, \mathbf{z_1}, \mathbf{z_2})$ without loss of generality. For any real patient data, $\bar{\mathbf{y}} = h(\bar{\mathbf{x}}, \bar{\mathbf{z}}_1, \bar{\mathbf{z}}_2) \sim p_{tar}(\mathbf{y})$, we can estimate its ground truth subtype, represented by $\bar{\mathbf{z}}_1$, through $g_1(\bar{\mathbf{y}}) = g_1\left(h(\bar{\mathbf{x}}, \bar{\mathbf{z}}_1, \bar{\mathbf{z}}_2)\right) \approx g_1\left(f(\bar{\mathbf{x}}, \bar{\mathbf{z}}_1, \bar{\mathbf{z}}_2)\right) \approx \bar{\mathbf{z}}_1$.

Supplementary Method 1.2: Modification of the GAN loss in the Gene-SGAN model: Motivation and Derivation.

In most GAN-based models[1,2], including Smile-GAN[3], the latent variables or noise variables are sampled from fixed distributions (e.g., gaussian or uniform distribution) predefined before the training process. The fixed latent distributions mostly do not affect the models' performances in generating realistic data. However, they are problematic if we use an inverse function to re-estimate the latent variables of the data. We focus on modifying the distribution of the major latent variable $\mathbf{z_1}$ since we mainly focus on using the inverse function g_1 to re-estimate $\mathbf{z_1}$, which defines the subtypes of interests.

As introduced in **Supplementary Method 1.1**, we assume the inverse consistency, $g_1\left(f(\mathbf{x}, \mathbf{z_1}, \mathbf{z_2})\right) \approx \mathbf{z_1}$, and equality in distributions, $p_{syn}(f(\mathbf{x}, \mathbf{z_1}, \mathbf{z_2})) \approx p_{tar}(\mathbf{y})$, after the training process. We can further derive that $p(g_1(\mathbf{y})) \approx p(g_1(f(\mathbf{x}, \mathbf{z_1}, \mathbf{z_2}))) \approx p(\mathbf{z_1})$, where $p(g_1(\mathbf{y}))$ is the distribution of patients' subtype memberships. Therefore, if we sample $\mathbf{z_1}$ from a uniform categorical distribution, the sizes of derived subtype memberships will also be balanced, which leads to bias when the ground-truth subtypes are not uniformly distributed.

In real applications, we can never acquire the exact equality of distributions, $p\left(f(\mathbf{x}, \mathbf{z_1}, \mathbf{z_2})\right) = p_{tar}(\mathbf{y})$. Thus, a slight deviation of the ground truth latent distribution from the uniform distribution does not significantly affect the models' performances. However, a severe imbalance in the sizes of ground truth subtypes does affect the Smile-GAN[3] and Gene-SGAN models if we sample the latent variable from the uniform distribution.

To resolve this issue, we make changes to the GAN loss function so that $\mathbf{z_1}$ is implicitly sampled from a parametrized distribution, $p_{\boldsymbol{\theta}_{\mathbf{z_1}}}(\mathbf{z_1})$, which is optimized to approximate the ground truth latent distribution. With $\mathbf{e_i}$ defined as a one-hot vector with one at the i_{th} dimension, we derive that:

$$L_{GAN}\left(\boldsymbol{\theta}_{\mathbf{D}}, \boldsymbol{\theta}_{\mathbf{f}}, \boldsymbol{\theta}_{\mathbf{z_1}}\right) = E_{\mathbf{y}\sim p_{tar}(\mathbf{y})}\left[\log\left(D(\mathbf{y})\right)\right] + E_{\mathbf{y}'\sim p_{syn}(\mathbf{y}')}\left[1 - \log\left(D(\mathbf{y}')\right)\right] \quad (1)$$

$$= E_{\mathbf{z_1}\sim p_{\boldsymbol{\theta}_{\mathbf{z_1}}}(\mathbf{z_1}), \mathbf{y}\sim p_{tar}(\mathbf{y})}\left[\log\left(D(\mathbf{y})\right)\right]$$

$$+ E_{z_1\sim p_{\theta_{z_1}}(z_1), z_2\sim p_{z_2}(z_2), x\sim p_{ref}(x)}\left[1-\log\left(D\left(f(\mathbf{x},\mathbf{z_1},\mathbf{z_2})\right)\right)\right] \quad (2)$$

$$= \sum_{i=1}^{M} p_{\theta_{z_1}}(\mathbf{e_i})\, E_{\mathbf{y}\sim p_{tar}(y)}\left[\log\left(D(\mathbf{y})\right)\right]$$

$$+ \sum_{i=1}^{M} p_{\theta_{z_1}}(\mathbf{e_i})\; E_{z_2\sim p_{z_2}(z_2), x\sim p_{ref}(x)}\left[1-\log\left(D\left(f(\mathbf{x},\mathbf{e_i},\mathbf{z_2})\right)\right)\right] \quad (3)$$

$$= M * \sum_{i=1}^{M} p_U(\mathbf{e_i}) p_{\theta_{z_1}}(\mathbf{e_i})\, E_{\mathbf{y}\sim p_{tar}(y)}\left[\log\left(D(\mathbf{y})\right)\right]$$

$$+ M * \sum_{i=1}^{M} p_U(\mathbf{e_i}) p_{\theta_{z_1}}(\mathbf{e_i})\; E_{z_2\sim p_{z_2}(z_2), x\sim p_{ref}(x)}\left[1-\log\left(D\left(f(\mathbf{x},\mathbf{e_i},\mathbf{z_2})\right)\right)\right] \quad (4)$$

$$= M * E_{\mathbf{y}\sim p_{tar}(y), \mathbf{z_1}\sim p_U(z_1)}\left[p_{\theta_{z_1}}(\mathbf{z_1})\log\left(D(\mathbf{y})\right)\right]$$

$$+ M * E_{\mathbf{z_1}\sim p_U(z_1), z_2\sim p_{z_2}(z_2), x\sim p_{ref}(x)}\left[p_{\theta_{z_1}}(\mathbf{z_1})\left(1-\log\left(D\left(f(\mathbf{x},\mathbf{z_1},\mathbf{z_2})\right)\right)\right)\right] \quad (5)$$

We derive the updated GAN loss function (5) from the basic objective function (1) presented in Goodfellow et al[1]. The updated loss function enables us to sample $\mathbf{z_1}$ from a uniform distribution but penalizes the losses with their probability under the distribution $p_{\theta_{z_1}}(\mathbf{z_1})$. In the training procedure, we only sample one value of $\mathbf{z_1}$ for each $\mathbf{x}$ per batch instead of taking the expectation over all M categories. Therefore, to penalize both terms equally, the same modification is also applied to the first term of the equation (5), in which $\mathbf{z_1}$ was not originally included.

Additionally, to prevent $p_{\theta_{z_1}}$ from converging to an extreme distribution (e.g., the probability of one category converges to zero), we added a regularization term that controls the distance between $p_{\theta_{z_1}}(\mathbf{z_1})$ and $p_U(\mathbf{z_1})$. Therefore, the final modified GAN loss function equals:

$$L_{GAN}\left(\boldsymbol{\theta_D}, \boldsymbol{\theta_f}, \boldsymbol{\theta_{z_2}}\right) = M * E_{\mathbf{y}\sim p_{tar}(y), \mathbf{z_1}\sim p_U(z_1)}\left[p_{\theta_{z_1}}(\mathbf{z_1})\log\left(D(\mathbf{y})\right)\right]$$

$$+ M * E_{\mathbf{z_1}\sim p_U(z_1), z_2\sim p_{z_2}(z_2), x\sim p_{ref}(x)}\left[p_{\theta_{z_1}}(\mathbf{z_1})\left(1-\log\left(D\left(f(\mathbf{x},\mathbf{z_1},\mathbf{z_2})\right)\right)\right)\right]$$

$$+ \kappa D_{KL}(p_U(\mathbf{z_1})|p_{\theta_{z_1}}(\mathbf{z_1})) \quad (6)$$

Supplementary Method 1.3: Derivation of the ELBO for the Gene Step.

In the Gene step, we attempted to approximate the intractable posterior distribution, $p_{\theta_h,\theta_{g_1}}(\mathbf{z_3}|\mathbf{v},\mathbf{y})$, by the variational distribution $q_{\theta_r}(\mathbf{z_3}|\mathbf{v},\mathbf{y})$. The KL divergence between $p_{\theta_h,\theta_{g_1}}(\mathbf{z_3}|\mathbf{v},\mathbf{y})$, and $q_{\theta_r}(\mathbf{z_3}|\mathbf{v},\mathbf{y})$ equals:

$$D_{KL}\left(q_{\theta_r}(\mathbf{z_3}|\mathbf{v},\mathbf{y})\middle|p_{\theta_h,\theta_{g_1}}(\mathbf{z_3}|\mathbf{v},\mathbf{y})\right)$$

$$= E_{z_3\sim q_{\theta_r}(\mathbf{z_3}|\mathbf{v},\mathbf{y})}[\log q_{\theta_r}(\mathbf{z_3}|\mathbf{v},\mathbf{y}) - \log p_{\theta_h,\theta_{g_1}}(\mathbf{z_3}|\mathbf{v},\mathbf{y})] \quad (7)$$

$$= E_{z_3\sim q_{\theta_r}(\mathbf{z_3}|\mathbf{v},\mathbf{y})}[\log q_{\theta_r}(\mathbf{z_3}|\mathbf{v},\mathbf{y}) - \log\frac{p_{\theta_h,\theta_{g_1}}(\mathbf{z_3},\mathbf{v},\mathbf{y})}{p(\mathbf{v},\mathbf{y})}] \quad (8)$$

Since the latent variable $\mathbf{z_3}$ characterizes genetic-specific variations, it is independent of $\mathbf{y}$, and the KL divergence can be further written as:

$$D_{KL}\left(q_{\theta_r}(\mathbf{z_3}|\mathbf{v},\mathbf{y})\middle|p_{\theta_h,\theta_{g_1}}(\mathbf{z_3}|\mathbf{v},\mathbf{y})\right)$$

$$= E_{\mathbf{z_3} \sim q_{\boldsymbol{\theta}_r}(\mathbf{z_3}|\mathbf{v},\mathbf{y})}[\log q_{\boldsymbol{\theta}_r}(\mathbf{z_3}|\mathbf{v},\mathbf{y}) - \log \frac{p_{\boldsymbol{\theta}_h,\boldsymbol{\theta}_{g_1}}(\mathbf{v}|\mathbf{z_3},\mathbf{y})p_{\mathbf{z_3}}(\mathbf{z_3})p(\mathbf{y})}{p(\mathbf{v}|\mathbf{y})p(\mathbf{y})}] \quad (9)$$

$$= E_{\mathbf{z_3} \sim q_{\boldsymbol{\theta}_r}(\mathbf{z_3}|\mathbf{v},\mathbf{y})}[\log q_{\boldsymbol{\theta}_r}(\mathbf{z_3}|\mathbf{v},\mathbf{y}) - \log p_{\boldsymbol{\theta}_h,\boldsymbol{\theta}_{g_1}}(\mathbf{v}|\mathbf{z_3},\mathbf{y}) - \log p_{\mathbf{z_3}}(\mathbf{z_3}) + \log p(\mathbf{v}|\mathbf{y})] \quad (10)$$

From this equation, we can further derive the ELBO for $p(\mathbf{v}|\mathbf{y})$:

$$\log p(\mathbf{v}|\mathbf{y}) \geq E_{\mathbf{z_3} \sim q_{\boldsymbol{\theta}_r}(\mathbf{z_3}|\mathbf{v},\mathbf{y})}[\log p_{\boldsymbol{\theta}_h,\boldsymbol{\theta}_{g_1}}(\mathbf{v}|\mathbf{z_3},\mathbf{y}) + \log \frac{p_{\mathbf{z_3}}(\mathbf{z_3})}{q_{\boldsymbol{\theta}_r}(\mathbf{z_3}|\mathbf{v},\mathbf{y})}] \quad (11)$$

Supplementary Method 2: Implementation details of Gene-SGAN

Supplementary Method 2.1: Model Architectures

The general architectures of the neural networks can be understood from **Figure 1b**. In the semi-synthetic and real data experiments, the dimensions of $\mathbf{z_2}$ and $\mathbf{z_3}$ are both set to five (i.e., $n_{\mathrm{z}_2} = n_{\mathrm{z}_3} = 5$); the dimension of the phenotypic features equals 144 or 145 in different cases. In the Phenotype step, the transformation function f utilizes an encoding-decoding structure. The REF features $\mathbf{x}$ and the concatenation of $\mathbf{z_1}$ and $\mathbf{z_2}$ are first mapped to two vectors with the dimension of 36, respectively. Their element-wise multiplication is then decoded to construct the synthesized TAR features $\mathbf{y}'$ with dimensions 144 or 145. The inverse mapping functions g_1 and g_2 share the same network, which maps the real/synthesized TAR features $\mathbf{y}/\mathbf{y}'$ to a vector with dimension (5+*M*). The last five elements represent the re-estimated $\mathbf{z_2}$, while a Softmax function is applied to the first *M* elements to derive *M* probability values. The discriminator D utilizes an encoding structure that maps the real/synthesized TAR features $\mathbf{y}/\mathbf{y}'$ to a vector with dimension two. In the Gene step, the concatenation of $\mathbf{z_3}$ and $\mathrm{g}_1(\mathbf{y})$ is mapped to an n_{genetic}-dimensional vector through a decoding neural network (i.e., the function h), which outputs the MAF of each SNP. The function r is backboned by an encoding neural network that maps the concatenated genetic and imaging data to a vector with dimension ten. More details of model architectures can be found in **Supplementary Table 1 and Table 2.**

Supplementary Method 2.2: Training Details

We set $\mu = 5$, $\lambda = 9$, and $\kappa = 0.1$ for all semi-synthetic and real data experiments. During the optimization procedure, the Phenotype and Gene steps were performed iteratively. Within the Phenotype step, we iteratively updated the parameters of the Discriminator ($\boldsymbol{\theta}_{\mathbf{D}}$), the latent distribution ($\boldsymbol{\theta}_{\mathbf{z_1}}$), as well as the transformation and reconstruction functions ($\boldsymbol{\theta}_{\mathbf{f}}$, $\boldsymbol{\theta}_{\mathbf{g_1}}$, and $\boldsymbol{\theta}_{\mathbf{g_2}}$). The detailed training procedure is revealed by **Supplementary Algorithm 1**. The ADAM optimizer[4] was used with a learning rate (*lr*) 2×10^{-4} for $\boldsymbol{\theta}_{\mathbf{D}}$, 2.5×10^{-5} for $\boldsymbol{\theta}_{\mathbf{z_1}}$, and 1×10^{-3} for $\boldsymbol{\theta}_{\mathbf{f}}$, $\boldsymbol{\theta}_{\mathbf{g_1}}$, and $\boldsymbol{\theta}_{\mathbf{g_2}}$. The learning rate of the Gene step (referred to as gene-lr) is a hyperparameter to be selected, as discussed in **Result** and **Method 6**. β_1 and β_2 for ADAM were set to be 0.5 and 0.999, respectively. Also, we performed gradient clipping for each iteration to avoid gradient explosions during the training process. The model was trained for at least 20000 iterations and saved until the Wasserstein Distance was smaller than 0.12 and the Alteration Quantity was smaller than 1/40 of the patients' sample sizes[3].

We performed weight clipping[5] to ensure the Lipschitz continuity of the transformation function and inverse functions. With Θ_{f}, Θ_{g_1}, and Θ_{g_2} denoting the weight spaces of f, g_1, and g_2, their compactness implies the Lipschitz continuity of the three functions. The compactness of Θ_{f}, Θ_{g_1}, and Θ_{g_2} was guaranteed by clapping these weight spaces into three closed boxes, $\Theta_{\mathrm{f}} = [-c_{\mathrm{f}}, c_{\mathrm{f}}]^{\mathrm{d}}$, $\Theta_{\mathrm{g}_1} = \left[-c_{\mathrm{g}_1}, c_{\mathrm{g}_1}\right]^{\mathrm{d}}$, and ,$\Theta_{\mathrm{g}_2} = \left[-c_{\mathrm{g}_2}, c_{\mathrm{g}_2}\right]^{\mathrm{d}}$. In the implementations, all clapping bounds were set to be 0.5 but can be further relaxed.

Supplementary Table 1: Network architectures of the function f

	Layer	Input Size	Bias Term	Leaky Relu α	Output Size
Encoder from $\mathbf{x}$	Linear1+Leaky-Relu	144 or 145	No	0.2	72
	Linear2+Leaky-Relu	72	No	0.2	36
Decoder from $\mathbf{z_1}$,$\mathbf{z_2}$	Linear1+Sigmoid	5+M	Yes	NA	36
Decoder to $\mathbf{y}'$	Linear1+Leaky-Relu	36	No	0.2	72
	Linear2+Leaky-Relu	72	No	0.2	144 or 145
	Linear3	144 or 145	No	NA	144 or 145

Supplementary Table 2: Network architecture of the functions D, $\mathbf{g_1}$&$\mathbf{g_2}$, h, r

	Layer	Input Size	Bias Term	Leaky Relu α	Output Size
Discriminator D	Linear1+Leaky-Relu	144 or 145	Yes	0.2	72
	Linear2+Leaky-Relu	72	Yes	0.2	36
	Linear3+Softmax	36	Yes	NA	2
Function g_1&g_2	Linear1+Leaky-Relu	144 or 145	Yes	0.2	144 or 145
	Linear2+Leaky-Relu	144 or 145	Yes	0.2	72
	Linear3+Leaky-Relu	72	Yes	0.2	36
	Linear4+Softmax	36	Yes	NA	5+M
Function h	Linear1+Leaky-Relu	5+M	Yes	0.2	(5+M)*2
	Linear2+Leaky-Relu	(5+M)*2	Yes	0.2	(5+M)*4
	0.2 Dropout+Linear3+Sigmoid	(5+M)*4	Yes	NA	n_{genetic}
Function r	Linear1+Leaky-Relu	n_{genetic}+144/145	Yes	0.2	(n_{genetic}+144/145)/2
	Linear2+Leaky-Relu	(n_{genetic}+144/145)/2	Yes	0.2	(n_{genetic}+144/145)/4
	Linear3+Leaky-Relu	(n_{genetic}+144/145)/4	Yes	0.2	10

Supplementary Algorithm 1: Gene-SGAN training procedure.

$\mathrm{l_c}$ represents cross entropy loss and $\mathbf{e_i}$ represents a one-hot vector with 1 at the $\mathrm{i_{th}}$ component.

while *not meeting stopping criteria or reaching max_epoch* **do**

for *all batches* $\{\mathbf{x^i}\}_{i=1}^{m}, \{\mathbf{y^i}\}_{i=1}^{m}, \{\mathbf{v^i}\}_{i=1}^{m}$ **do**

Perform Phenotype Step with GAN:

Sample m integers $\{a^{\mathrm{i}}\}_{\mathrm{i}=1}^{m}$ *with* $a^{\mathrm{i}} \sim \text{discrete-U}(1, M)$ *and let* $\mathbf{z_1^i} = \mathbf{e}_{a^{\mathrm{i}}}$

Sample m vectors $\{\mathbf{z_2^i}\}_{\mathrm{i}=1}^{m}$ *from a multivariate uniform distribution with* $\mathbf{z_2^i} \sim \mathrm{U}[0,1]^{n_{z_2}}$

Update weights of discriminator D: Use ADAM to update $\boldsymbol{\theta_D}$ with gradient:

$\nabla_{\boldsymbol{\theta_D}} \frac{1}{m} \sum_{\mathrm{i}=1}^{m} [\mathrm{p}_{\boldsymbol{\theta}_{\mathbf{z_1}}}(\mathbf{z_1^i})(\mathrm{l_c}(\mathrm{D}(\mathbf{y^i}), \mathbf{e_1}) + \mathrm{l_c}(\mathrm{D}(\mathrm{f}(\mathbf{x^i}, \mathbf{z_1^i}, \mathbf{z_2^i}), \mathbf{e_0})))]$

Update weights of functions f, $\mathbf{g_1}$, and $\mathbf{g_2}$: Use ADAM to update $\boldsymbol{\theta_f}, \boldsymbol{\theta_{g_1}}, \boldsymbol{\theta_{g_2}}$ with gradient:

$\nabla_{\boldsymbol{\theta_f}} \frac{1}{m} \sum_{\mathrm{i}=1}^{m} [(\mathrm{p}_{\boldsymbol{\theta}_{\mathbf{z_1}}}(\mathbf{z_1^i}) \mathrm{l_c}\big(\mathrm{D}(\mathrm{f}(\mathbf{x^i}, \mathbf{z_1^i}, \mathbf{z_2^i}), \mathbf{e_1}\big) + \lambda(\mathrm{l_c}(\mathrm{g_1}\big(\mathrm{f}(\mathbf{x^i}, \mathbf{z_1^i}, \mathbf{z_2^i}), \mathbf{z_1^i}\big) + \| \mathrm{g_2}\left(\mathrm{f}(\mathbf{x^i}, \mathbf{z_1^i}, \mathbf{z_2^i})\right) - \mathbf{z_2^i} \|_2) + \mu \big\| \mathrm{f}(\mathbf{x^i}, \mathbf{z_1^i}, \mathbf{z_2^i}) - \mathbf{x_i} \big\|_1)]$

$\nabla_{\boldsymbol{\theta}_{\mathbf{g_1}}} \frac{1}{m} \sum_{i=1}^{m} [l_c(g_1(f(\mathbf{x}^i, \mathbf{z}_1^i, \mathbf{z}_2^i), \mathbf{z}_1^i))]$

$\nabla_{\boldsymbol{\theta}_{\mathbf{g_2}}} \frac{1}{m} \sum_{i=1}^{m} [\| g_2\left(f(\mathbf{x}^i, \mathbf{z}_1^i, \mathbf{z}_2^i)\right) - \mathbf{z}_2^i \|_2]$

Update weights of the parametrized distribution $\mathbf{p}_{\boldsymbol{\theta}_{\mathbf{z_1}}}(\mathbf{z_1})$: Use ADAM to update $\boldsymbol{\theta}_{\mathbf{z_1}}$ with gradient:

$\nabla_{\boldsymbol{\theta}_{\mathbf{z_1}}} \frac{1}{m} \sum_{i=1}^{m} (p_{\boldsymbol{\theta}_{\mathbf{z_1}}}(\mathbf{z}_1^i) l_c(D(f(\mathbf{x}^i, \mathbf{z}_1^i, \mathbf{z}_2^i), \mathbf{e_1}))) + \kappa D_{KL}(p_U(\mathbf{z_1}) | p_{\boldsymbol{\theta}_{\mathbf{z_1}}}(\mathbf{z_1}))$

Perform weight clipping:

$(\boldsymbol{\theta}_{\mathbf{f}}, \boldsymbol{\theta}_{\mathbf{g_1}}, \boldsymbol{\theta}_{\mathbf{g_2}}) = \text{clip}((\boldsymbol{\theta}_{\mathbf{f}}, \boldsymbol{\theta}_{\mathbf{g_1}}, \boldsymbol{\theta}_{\mathbf{g_2}}), -c, c)$

Perform Gene Step with VI:

Sample m latent vectors $\{\mathbf{z}_3^i\}_{i=1}^{m}$ *from m posterior distributions* $q_{\boldsymbol{\theta}_{\mathbf{r}}}(\mathbf{z}_3^i | \mathbf{v}^i, \mathbf{y}^i)$ *with the reparameterization trick.*

Update weights of functions $\mathbf{g_1}$, h, and r: Use ADAM to update $\boldsymbol{\theta}_{\mathbf{r}}, \boldsymbol{\theta}_{\mathbf{h}}, \boldsymbol{\theta}_{\mathbf{g_1}}$ with gradient:

$\nabla_{\boldsymbol{\theta}_{\mathbf{r}}} \frac{1}{m} \sum_{i=1}^{m} [\log p_{\boldsymbol{\theta}_{\mathbf{h}}, \boldsymbol{\theta}_{\mathbf{g_1}}}(\mathbf{v}^i | \mathbf{z}_3^i, \mathbf{y}^i) + \log p_{\mathbf{z_3}}(\mathbf{z}_3^i) - \log q_{\boldsymbol{\theta}_{\mathbf{r}}}(\mathbf{z}_3^i | \mathbf{v}^i, \mathbf{y}^i)]$

$\nabla_{\boldsymbol{\theta}_{\mathbf{h}}} \frac{1}{m} \sum_{i=1}^{m} [\log p_{\boldsymbol{\theta}_{\mathbf{h}}, \boldsymbol{\theta}_{\mathbf{g_1}}}(\mathbf{v}^i | \mathbf{z}_3^i, \mathbf{y}^i)]$

$\nabla_{\boldsymbol{\theta}_{\mathbf{g_1}}} \frac{1}{m} \sum_{i=1}^{m} [\log p_{\boldsymbol{\theta}_{\mathbf{h}}, \boldsymbol{\theta}_{\mathbf{g_1}}}(\mathbf{v}^i | \mathbf{z}_3^i, \mathbf{y}^i)]$

end

end

Supplementary Method 3: Implementation details of model comparisons in the semi-synthetic experiments

For the Gene-SGAN model, we selected the optimal gene-lr using the CV procedure (**Method 6**). Other hyperparameters and network architectures were fixed as introduced in **Supplementary Method 2.2**. For the other compared methods, the implementation details were introduced in **Supplementary Table 3**. For Smile-GAN, MSC, CCA, and Deep-CCA, we ran models with different combinations of hyperparameters and reported the best performances. For CCA and Deep-CCA, the Kmeans algorithm with n_init=200 and max_iter=500 was further applied to the outputted embeddings to derive cluster memberships.

Supplementary Table 3: Implementation details of the six compared methods.

Model	Package	Features	Hyperparameters
Smile-GAN	SmileGAN 0.1.2	Imaging	lam = [7,8,9,10,11]; mu = [3,4,5,6,7];
Kmeans	scikit-learn 0.24.2	Imaging + Genetic	n_init = 200; max_iter=500;
MKmeans	mvlearn 0.5.0[6]	Imaging + Genetic	n_init = 200; max_iter=500;<br>patience = 30;
MSC	mvlearn 0.5.0	Imaging + Genetic	n_init = 200; max_iter = 500;<br>affinity = 'nearest neighbors';<br>n_neighbors = [30,35,40,45,50]
CCA	scikit-learn 0.24.2	Imaging + Genetic	n_components = [3,4,5,6,7,8,9,10];<br>max_iter = 500
Deep-CCA	mvlearn 0.5.0	Imaging + Genetic	n_components = [3,4,5,6,7,8,9,10];<br>hidden_layer_size = [128,256,512,1024];<br>n_hidden_layers = 2;<br>epoch_num = 1000;

Supplementary Method 4: Replication analyses for SNP-subtype associations

In the split-sampled experiments on the hypertension dataset, we tested the reproducibility of the SNP-subtype associations found among hypertensive patients in the discovery set. Specifically, we filtered out all significantly associated SNPs after Bonferroni correction in the discovery set. Then, for each SNP, we constructed two *M*-dimensional vectors, one for the discovery set and one for the replication set. The i_{th} component of the vector equals the MAF of the SNP within the i_{th} subtype. Pearson's correlation between two vectors was calculated for each SNP to analyze consistencies in subtype-wise relationships. Lastly, we used the replication set to retest SNPs with correlations greater than 0.5. We reported the number of significantly associated SNPs after B-H and Bonferroni corrections, respectively.

Supplementary Method 5: Adjusted N-Asso-SNPs for selecting the optimal number of clusters

We used N-Asso-SNPs as a metric to select hyperparameters while fixing the number of clusters. However, this measure tended to increase with the number of clusters without adjustments. Therefore, we proposed an adjusted version of N-Asso-SNPs, considering chance associations, for optimal cluster selection. The observed N-Asso-SNPs were obtained through cross-validation (**Method 6**). To establish a null distribution, we conducted a 100-iteration permutation test by randomly shuffling subtype memberships before deriving the N-Asso-SNPs. Subtracting the 100 null N-Asso-SNPs from the observed value yielded a distribution of adjusted N-Asso-SNPs (**Supplementary Figure 1**).

Supplementary Method 6: **Examination of genetic and phenotypic variations captured by $\mathbf{z_2}$ and $\mathbf{z_3}$.**

We examined the latent variables $\mathbf{z_2}$ and $\mathbf{z_3}$ derived by Gene-SGAN on the MCI/AD dataset with M=4. After the training process, the inverse mapping function g_2 is applied to patients' imaging ROIs to estimate their $\mathbf{z_2}$ variables. In addition, the function r is applied to concatenated ROIs and AD-associated SNPs to derive the posterior distributions of $\mathbf{z_3}$. The mean of each derived distribution is used as an estimated $\mathbf{z_3}$ for each participant. We concatenated $\mathbf{z_2}$ across all 50 trained models (**Method 7**) for each participant and derived the first five principal components as z_2PC1-z_2PC5. The first five principal components of $\mathbf{z_3}$, z_3PC1-z_3PC5, were derived through the same procedure.

Supplementary Method 6.1: Associations of $\mathbf{z_2}$ and $\mathbf{z_3}$ with imaging volumetric measures in MCI/AD.

We performed voxel-based morphometry analyses through Nilearn[7] and gray matter RAVENS maps[8]. Specifically, we fitted a linear regression model with voxel-wise volumetric measures as dependent variables and each PC as an independent variable, adjusting for covariates including age, gender, ICV, and probabilities of subtypes. Two-tailed t-tests were performed to test associations between each PC and imaging volumetric measures. The Benjamin-Hochberg method was used to correct for multiple comparisons.

Supplementary Method 6.2: Associations of $\mathbf{z_2}$ and $\mathbf{z_3}$ with AD-associated SNPs.

To test associations between each PC of $\mathbf{z_2}$ or $\mathbf{z_3}$ and AD-associated SNPs, we fitted a linear regression model with SNPs as dependent variables and each PC as an independent variable, adjusting for covariates including age, gender, ICV, and probabilities of subtypes. Two-tailed t-tests were performed to test associations between each PC and each SNP, and the Bonferroni method was used to correct for multiple comparisons.

Supplementary Method 7: Reproducibility analyses of subtype memberships and latent variables

Supplementary Method 7.1: Reproducibility analyses of AD-related subtypes

We evaluated the reproducibility of the subtypes (encoded by $\mathbf{z_1}$) and latent variables, $\mathbf{z_2}$ and $\mathbf{z_3}$, derived on the MCI/AD datasets using nested cross-validation (CV) and external reference experiments.

Nested cross-validation: we performed a 5-fold nested CV to evaluate the replicability of the derived latent variables ($\mathbf{z_1}$-$\mathbf{z_3}$). Specifically, we partitioned both the PT and HC data into five folds. In each iteration of the outer loop, we used 4 folds of data (80%), and performed a 20% holdout CV with 50 repetitions as the inner loop. The consensus of models identified subtypes ($\mathbf{z_1}$), z_2PCs, and z_3PCs (following **Supplementary Method 6**). Agreement of subtypes among the five consensus results was measured using Adjusted Mutual Information (AMI). Pearson's correlations of the first three PCs quantified agreements for $\mathbf{z_2}$ and $\mathbf{z_3}$.

External reference experiment: We randomly selected 1000 healthy control subjects aged over 60 years from the UKBB study as an independent reference group. In the first experiment, we applied the trained models with *M*=4 to the ADNI MCI/AD participants, standardized with respect to the UKBB reference group. In the second experiment, we retrained the model with *M*=4 following the process in **Method 7**, with MCI/AD participants as the target group but UKBB participants as the reference group. Replicability was assessed by comparing new subtype memberships with the original four subtypes using AMI.

Supplementary Method 7.2: Reproducibility analyses of hypertension-related subtypes

We evaluated the reproducibility of the subtypes and latent variables derived on the hypertension datasets using nested CV (**same as Supplementary Method 7.1**) and stratified split-sampled experiments.

Stratified split-sampled experiments: Different from the split-sampled experiments described in **Method 7**, we additionally half split the HC group to further investigate the impact of a different reference group in model application and training. As detailed in **Supplementary Table 5**, we trained and applied the Gene-SGAN model with different combinations of reference groups (non-hypertensive participants) and target groups (hypertensive participants) from Splits 1 and 2, which helped explore results replicability across the following scenarios: (1) baseline case; (2) an independent reference group in model application; (3) a different reference group in model training; (4) different target and reference groups in model training.

Supplementary Note 1: Gene-SGAN produces reproducible subtypes and latent variables

Supplementary Note 1.1: Gene-SGAN produces reproducible hypertension-related subtypes and latent variables.

Through 5-fold nested CV, we observed prominent AMIs among subtypes and notable correlations among PCs of $\mathbf{z_2}$ and $\mathbf{z_3}$ (**Supplementary Table 6**). These results indicate that the Gene-SGAN model consistently produces reproducible latent variables on the hypertension datasets. Additionally, in our stratified split-sampled experiments, we found that utilizing an independent reference group in model application had minimal impact on subtype memberships (AMI = 0.865). This result aligns with the high generalizable testing accuracy observed in semi-synthetic experiments (**Supplementary Figure 1**). Employing a completely different reference group in model training also resulted in highly consistent subtyping (AMI = 0.728). Moreover, substituting the target group led to lower but still observable agreements in subtyping results (AMI = 0.509) (**Supplementary Table 5**).

Supplementary Note 1.2: Gene-SGAN produces reproducible AD-related subtypes and latent variables.

Compared with the replicability of results on the hypertension dataset, AD-related subtypes and z_2PCs have generally lower AMIs and correlations in nested CV (**Supplementary Table 4**). The much smaller sample sizes might contribute to the moderately lower replicability. The higher correlations observed in z_3PCs can be attributed to higher proportions of associated candidate SNPs on the same chromosome (Chr19). Additionally, consistent with results in semi-synthetic experiments, an external reference group from UKBB in model application did not yield significant changes in subtyping (AMI=0.777). Comparatively, when utilized in model retraining, it did lead to less agreeable results (AMI=0.540). Nevertheless, given the level of reproducibility in the nested CV, the impact of an external reference group in model retraining is minimal, accounting for the sample size and model randomness.

Supplementary Note 1.3: Gene-SGAN produces consistent subtypes and latent variables across different scales.

Increasing the number of clusters (M=3 to 5) for both MCI/AD and hypertension did not lead to changes in the majority of participants' subtypes while still maintaining reproducible imaging signatures (**Supplementary Figure 2 and 3**). Quantitatively, there were satisfactory agreements in subtype membership across three different scales, as measured by AMI (**Supplementary Table 7 and 8**). As expected, the lowest AMI values were observed between M=3 and M=5 due to the larger differences in scale. Moreover, we observed notable correlations among z_2 and z_3 PCs across different scales, further validating the reproducibility of Gene-SGAN's results.

Supplementary Table 4: Reproducibility of AD-related subtypes in nested cross-validation

Nested CV	AMI of Subtypes (z_1)	Correlation of z_2	Correlation of z_3
***M*=3**	0.540±0.078	0.691±0.077	0.884±0.053
***M*=4**	0.488±0.051	0.588±0.053	0.885±0.054
***M*=5**	0.459±0.041	0.542±0.110	0.896±0.037

Supplementary Table 5: Reproducibility of hypertension-related subtypes in stratified split-sampled experiments

Training Set		Test Set		AMI compared to Baseline
**Reference**	**Target**	**Reference**	**Target**	
Split1	Split1	Split1	Split2	1.000 (Baseline)
Split1	Split1	**Split2**	Split2	0.865
**Split2**	Split1	Split1	Split2	0.728
**Split2**	**Split2**	Split1	Split2	0.509

Supplementary Table 6: Reproducibiligy of hypertension-related subtypes in nested cross-validation

Nested CV	AMI of Subtypes (z_1)	Correlation of z_2	Correlation of z_3
***M*=3**	0.606±0.038	0.620±0.086	0.632±0.051
***M*=4**	0.643±0.023	0.687±0.081	0.613±0.042
***M*=5**	0.612±0.025	0.621±0.075	0.560±0.069

Supplementary Table 7: Consistency of AD-related subtypes and latent variables across different model scales *M*.

	AMI of Subtypes (z_1)	Correlation of z_2	Correlation of z_3
***M*=3 vs *M*=4**	0.505	0.656	0.960
***M*=3 vs *M*=5**	0.468	0.501	0.882
***M*=4 vs *M*=5**	0.618	0.708	0.941

Supplementary Table 8: Consistency of hypertension-related subtypes and latent variables across different model scales *M*.

	AMI of Subtypes (z_1)	Correlation of z_2	Correlation of z_3
***M*=3 vs *M*=4**	0.509	0.630	0.567
***M*=3 vs *M*=5**	0.441	0.518	0.525
***M*=4 vs *M*=5**	0.653	0.504	0.515

Supplementary Figure 1: Gene-SGAN identifies the simulated ground truth in semi-synthetic experiments

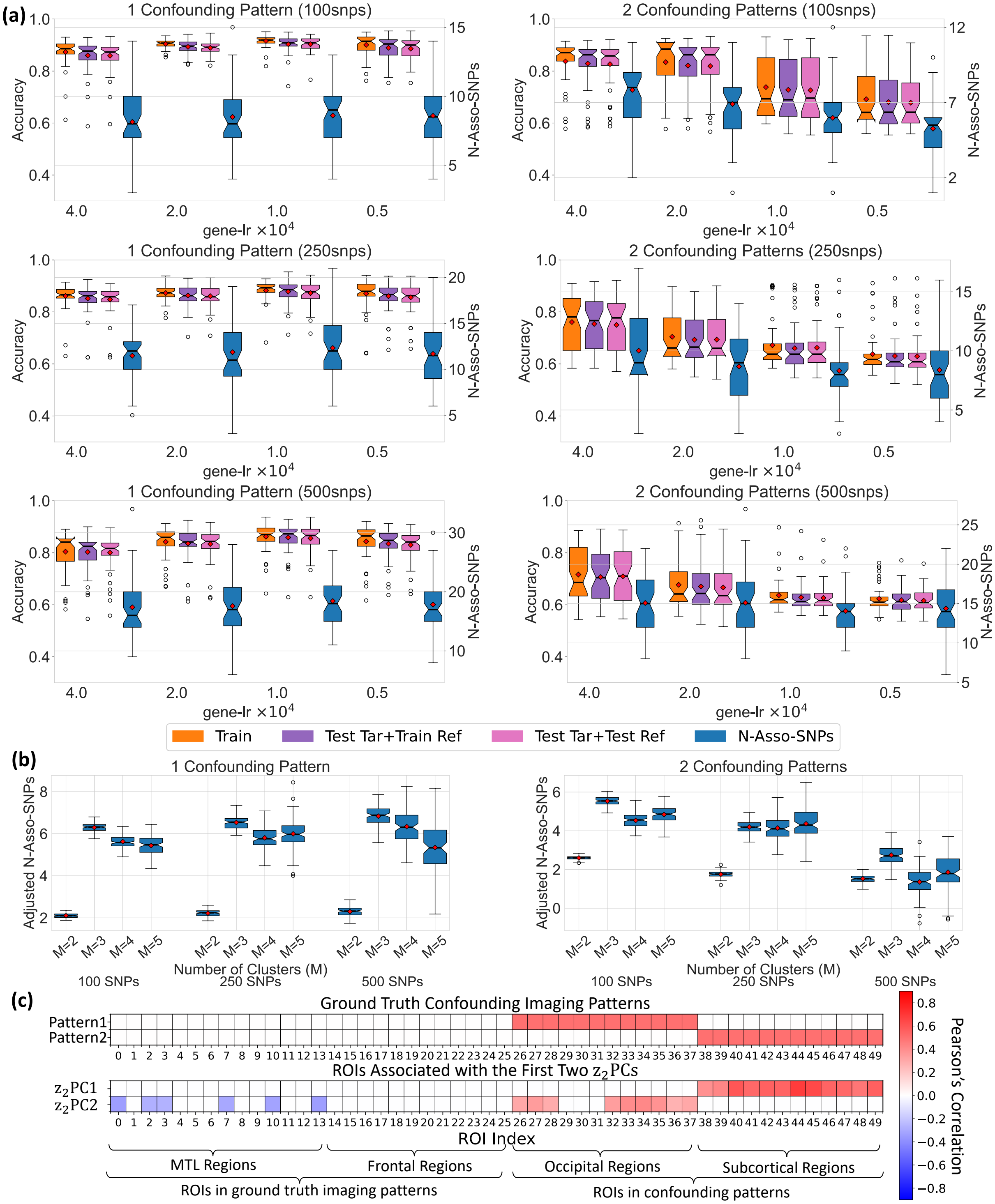

(a) Gene-SGAN shows robust generalizability to test data. With different hyperparameter (gene-lr) settings, SNP data dimensions, and levels of imaging confounder, Gene-SGAN consistently achieves comparable clustering accuracies on the training and test sets. Standardizing the target (Tar) features with respect to the test reference (Ref) group did not result in decreased clustering accuracies. In addition, N-Asso-SNPs, the parameter selection metric, was positively correlated with the clustering accuracies, serving as an appropriate metric for suggesting the optimal hyperparameter

in real data applications. The left axis reveals the scale of clustering accuracies on the training and test sets (Orange and Purple). The right axis reveals the scale of N-Asso-SNPs (blue). (b) Adjusted N-Asso-SNPs could suggest the ground truth number of clusters in most cases and thus could be potentially utilized as a metric for the cluster number selections. We set the number of clusters M to 2-5 and trained the Gene-SGAN model on six different semi-synthetic datasets that have three ground truth subtypes. (c) The first two PCs of $\mathbf{z_2}$ are primarily correlated with two simulated confounding imaging patterns without genetic associations. For presenting the ground truth, we identically colored the ROIs included in the two confounding imaging patterns. For visualization of strong ROI-z_2PC associations, we only colored ROIs with absolute correlations>0.25. MTL: medial temporal lobe. (Centerline, median; red marker: mean; box limits, upper and lower quartiles; whiskers, 1.5× interquartile range; points, outliers)

Supplementary Figure 2: Gene-SGAN provides consistent and refined multiscale imaging subtypes related to AD.

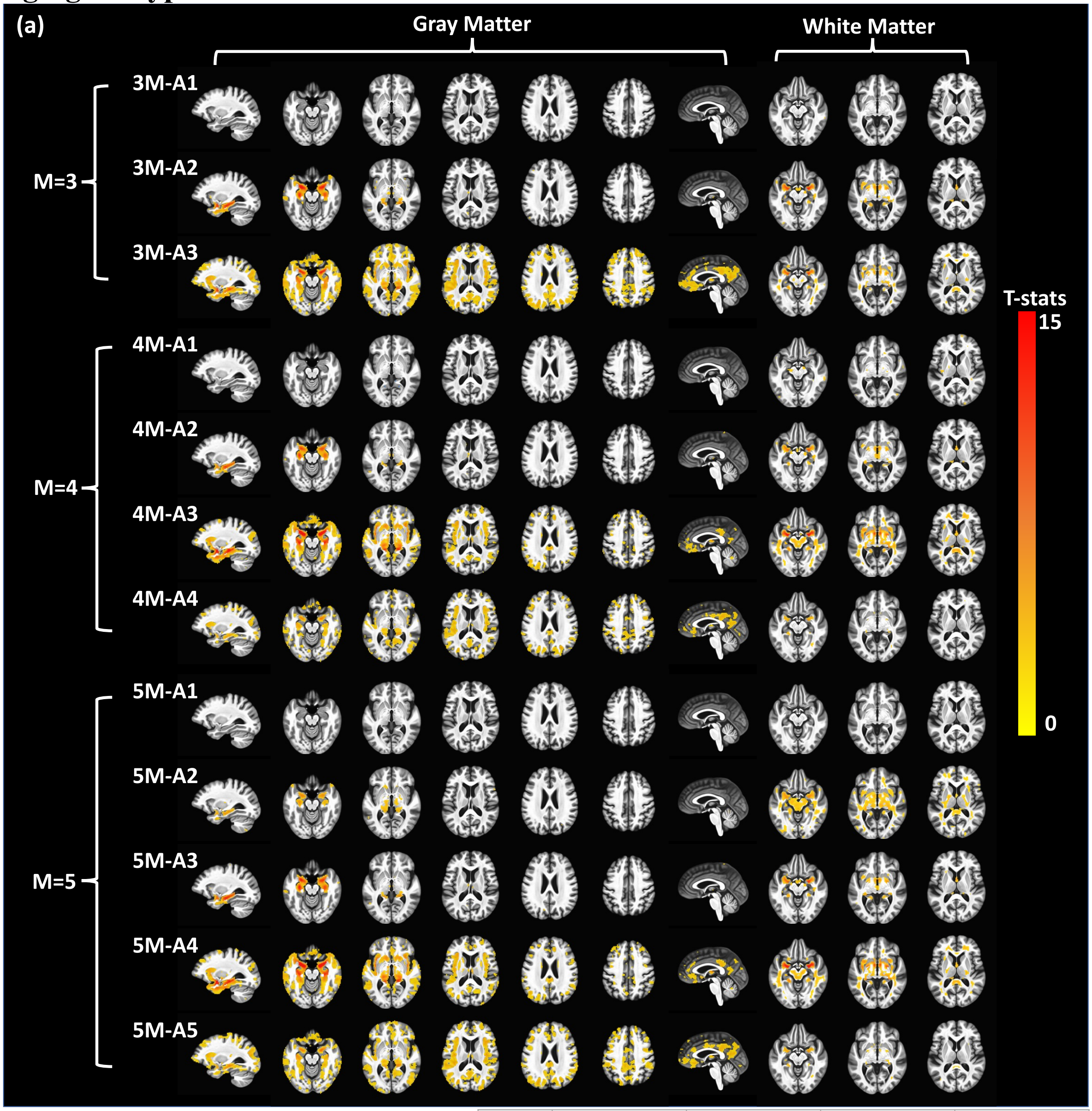

(b)

	3M-A1	3M-A2	3M-A3
**4M-A1**	**311 (100%)**	0	0
**4M-A2**	22 (11.2%)	**169 (85.8%)**	6 (3.0%)
**4M-A3**	2 (0.7%)	108 (38.4%)	171 (60.9%)
**4M-A4**	81 (29.8%)	8 (3.0%)	**183 (67.2%)**

	4M-A1	4M-A2	4M-A3	4M-A4
**5M-A1**	**247 (85.2%)**	15 (5.2%)	0	28 (9.6%)
**5M-A2**	59 (50.4%)	36 (30.8%)	20 (17.1%)	2 (1.7%)
**5M-A3**	2 (1.2%)	**144 (83.2%)**	15 (8.7%)	12 (6.9%)
**5M-A4**	0	2 (0.9%)	**230 (97.0%)**	5 (2.1%)
**5M-A5**	3 (1.2%)	0	16 (6.6%)	**225 (92.2%)**

a Voxel-wise group comparisons (two-sided t-test) were performed between the HC group (i.e., cognitively normal participants) and participants dominated by each AD-related subtype derived with different scales *M*=3-5. False discovery rate (FDR) correction for multiple comparisons with a p-value threshold of 0.05 was applied. xM-Ay refers to the y-th AD-related subtype derived by Gene-SGAN with the number of clusters *M*=x. Warmer color denotes more brain atrophy in the subtype versus HC. **b** Tables show the reallocation of participants in refined subtypes. For instance, the data in the first cell of the right table, 247 (85.2%), can be interpreted as 247 participants in 5M-A1 coming from 4M-A1, which is 85.2% of all participants in 5M-A1. The highest fraction in each row is bolded.

Supplementary Figure 3: Gene-SGAN provides consistent and refined multiscale imaging subtypes related to hypertension.

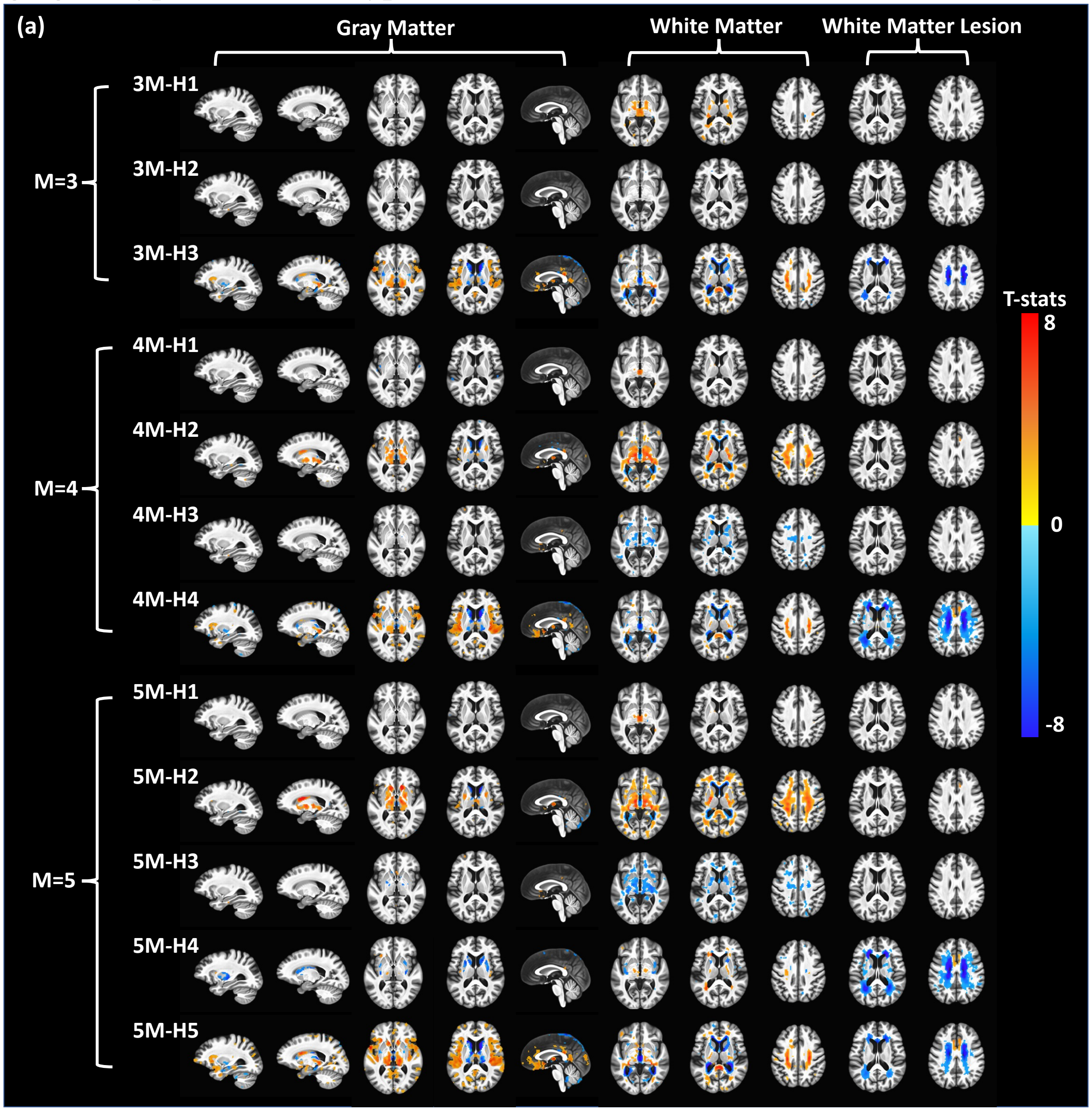

(b)

	3M-H1	3M-H2	3M-H3
**4M-H1**	**4638 (94.1%)**	291 (5.9%)	0
**4M-H2**	1173 (30.8%)	**1938 (50.9%)**	699 (18.3%)
**4M-H3**	89 (1.6%)	**5055 (91.9%)**	356 (6.5%)
**4M-H4**	0 (29.8%)	109 (5.0%)	**2066 (95.0%)**

	4M-H1	4M-H2	4M-H3	4M-H4
**5M-H1**	**4434 (95.3%)**	138 (3.0%)	80 (1.7%)	0
**5M-H2**	274 (7.7%)	**3087 (87.1%)**	174 (4.9%)	8 (0.2%)
**5M-H3**	216 (4.3%)	29 (0.6%)	**4731 (93.8%)**	68 (1.3%)
**5M-H4**	5 (0.4%)	373 (27.8%)	368 (27.4%)	**595 (44.4%)**
**5M-H5**	0 (1.2%)	183 (10.0%)	147 (8.0%)	**1504 (82.0%)**

a Voxel-wise statistical comparisons (two-sided *t*-test) were performed between the HC group (non-hypertensive participants) and participants dominated by each hypertension-related subtype derived with different scales M=3-5. FDR correction for multiple comparisons with a p-value threshold of 0.05 was applied. xM-Hy refers to the y-th hypertension-related subtype derived by Gene-SGAN with the number of clusters M=x. Warmer color denotes brain atrophy (i.e., HC > subtype), and cooler color represents larger tissue volume (i.e., subtype > HC). **b** Tables show the reallocation of participants in refined subtypes. For instance, data in the first cell of the right table, 4434 (95.3%), can be interpreted as 4434 participants in 5M-H1 coming from 4M-H1, which is 95.3% of all participants in 5M-H1. The highest fraction in each row is bolded.

Supplementary Figure 4: z_2 and z_3 variables capture additional non-linked imaging-specific and genetic-specific variations among MCI/AD participants.

(a)

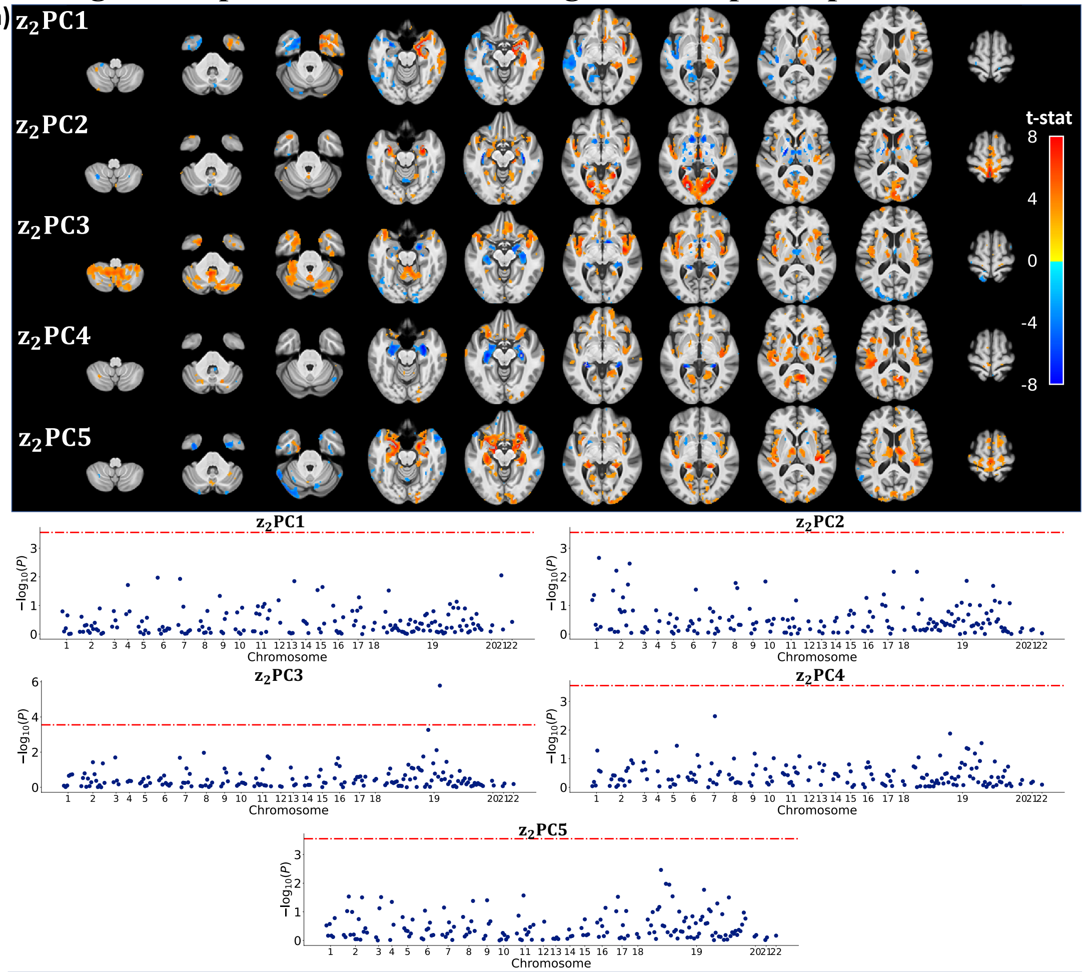

(b)

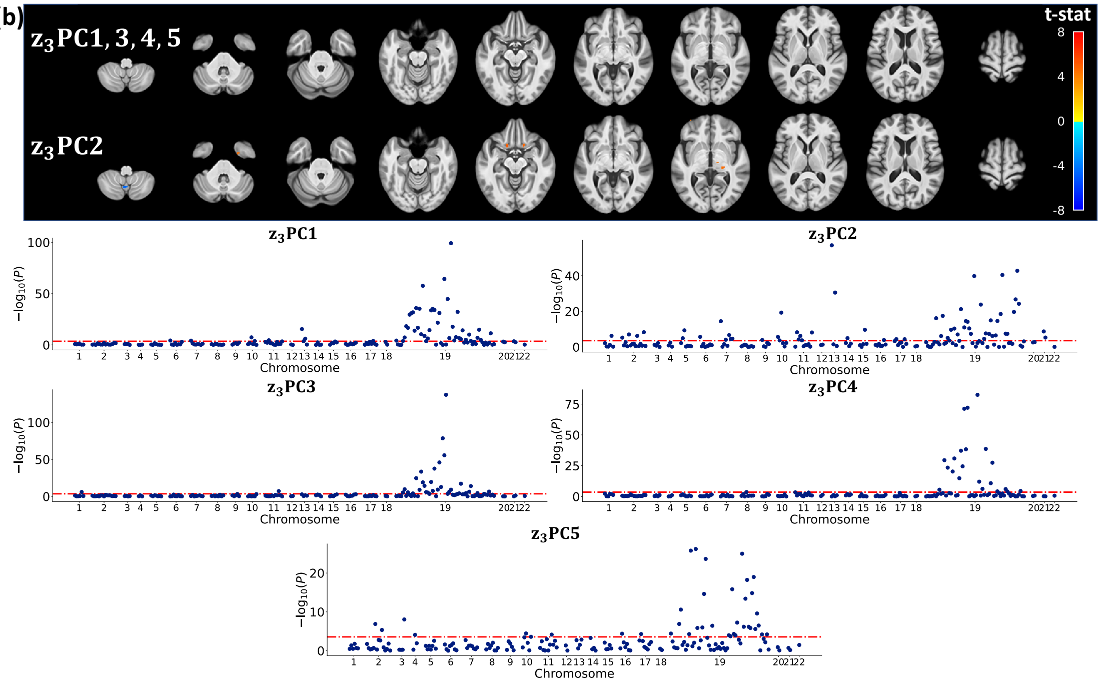

a The first five PCs of $\mathbf{z_2}$ primarily capture additional non-genetically associated variations in imaging features, including asymmetry of MTL atrophy (z_2PC1), atrophy in the cerebellum and cuneus (z_2PC2 and z_2PC3), as well as the severity (stages) of brain changes in regions associated with four AD-related subtypes (z_2PC4 and z_2PC5). In contrast, only one PC, z_2PC3, has a significant association with a single SNP. **b** The first five PCs of $\mathbf{z_3}$ mainly capture additional genetic-specific variations without significantly associated imaging patterns. Only z_3PC2 shows very mild associations with volume changes in the basal forebrain. For voxel-based morphometry analyses, FDR correction was performed to adjust for multiple comparisons with a p-value threshold of 0.05. Warmer color denotes a positive association between voxel-wise volumetric measures and PCs, and cooler color denotes a negative association. In the Manhattan plots, the dashed lines denote the p-value threshold of 0.05 after adjusting for multiple comparisons via the Bonferroni method.

Supplementary Figure 5: Gene-SGAN identifies hypertension-related subtypes with replicable imaging signatures in the discovery and replication sets.

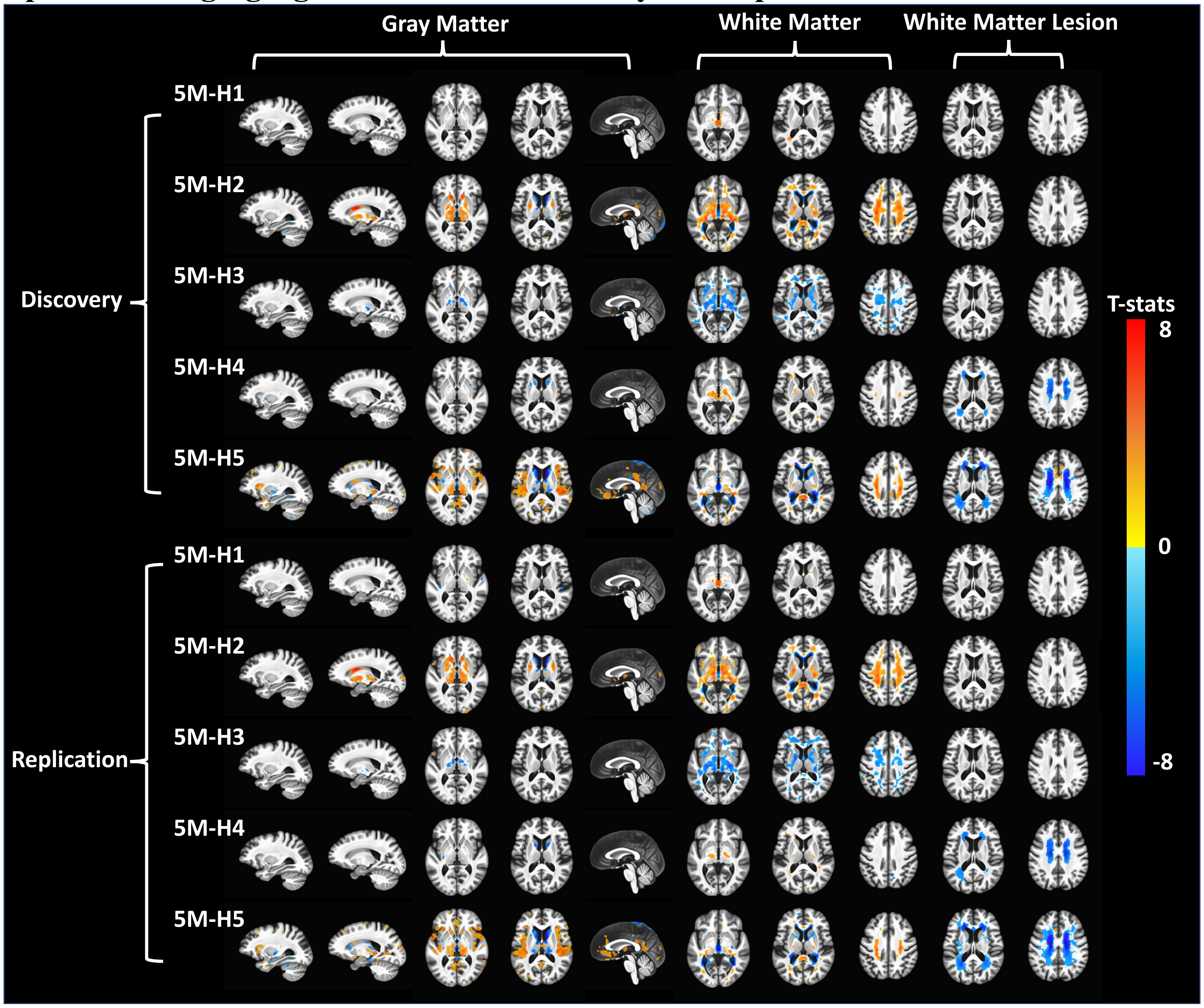

Voxel-wise statistical comparisons (two-sided *t*-test) were performed between the HC group (non-hypertensive participants) and participants dominated by each hypertension-related subtype derived in split-sampled experiments. FDR correction for multiple comparisons with a p-value threshold of 0.05 was applied. xM-Hy refers to the y-th hypertension-related subtype with the number of clusters *M*=x. Warmer color denotes brain atrophy (i.e., HC > subtype), and cooler color represents larger tissue volume (i.e., subtype > HC).

Supplementary References

1 Goodfellow, I. *et al.* Generative Adversarial Networks. *Advances in Neural Information Processing Systems* **3**, doi:10.1145/3422622 (2014).

2 Chen, X. *et al. InfoGAN: Interpretable Representation Learning by Information Maximizing Generative Adversarial Nets*. (2016).

3 Yang, Z. *et al.* A deep learning framework identifies dimensional representations of Alzheimer's Disease from brain structure. *Nature Communications* **12**, doi:10.1038/s41467-021-26703-z (2021).

4 Kingma, D. & Ba, J. Adam: A Method for Stochastic Optimization. *International Conference on Learning Representations* (2014).

5 Arjovsky, M., Chintala, S. & Bottou, L. in *Proceedings of the 34th International Conference on Machine Learning* Vol. 70 (eds Precup Doina & Teh Yee Whye) 214--223 (PMLR, Proceedings of Machine Learning Research, 2017).

6 Perry, R. *et al. mvlearn: Multiview Machine Learning in Python*. (2020).

7 Abraham, A. *et al.* Machine learning for neuroimaging with scikit-learn. *Front Neuroinform* **8**, 14, doi:10.3389/fninf.2014.00014 (2014).

8 Davatzikos, C., Genc, A., Xu, D. & Resnick, S. M. Voxel-based morphometry using the RAVENS maps: methods and validation using simulated longitudinal atrophy. *Neuroimage* **14**, 1361-1369, doi:10.1006/nimg.2001.0937
S1053-8119(01)90937-1 [pii] (2001).
