# Supplementary material for: Gene-SGAN: discovering disease subtypes with imaging and genetic signatures via multi-view weakly-supervised deep clustering
Source: Nat Commun. 2024 Jan 8;15:354. doi: 10.1038/s41467-023-44271-2 (PMC10774282; doi:10.1038/s41467-023-44271-2)
Supplement: Supplementary file 4 — Reporting Summary [file 41467_2023_44271_MOESM4_ESM.pdf]

nature portfolio

Corresponding author(s): Christos Davatzikos

Last updated by author(s): Nov 1, 2023

Reporting Summary

Nature Portfolio wishes to improve the reproducibility of the work that we publish. This form provides structure for consistency and transparency in reporting. For further information on Nature Portfolio policies, see our Editorial Policies and the Editorial Policy Checklist.

Statistics

For all statistical analyses, confirm that the following items are present in the figure legend, table legend, main text, or Methods section.

n/a	Confirmed	
☐	☒	The exact sample size (n) for each experimental group/condition, given as a discrete number and unit of measurement
☐	☒	A statement on whether measurements were taken from distinct samples or whether the same sample was measured repeatedly
☐	☒	The statistical test(s) used AND whether they are one- or two-sided<br>*Only common tests should be described solely by name; describe more complex techniques in the Methods section.*
☐	☒	A description of all covariates tested
☐	☒	A description of any assumptions or corrections, such as tests of normality and adjustment for multiple comparisons
☐	☒	A full description of the statistical parameters including central tendency (e.g. means) or other basic estimates (e.g. regression coefficient) AND variation (e.g. standard deviation) or associated estimates of uncertainty (e.g. confidence intervals)
☐	☒	For null hypothesis testing, the test statistic (e.g. F, t, r) with confidence intervals, effect sizes, degrees of freedom and P value noted<br>*Give P values as exact values whenever suitable.*
☒	☐	For Bayesian analysis, information on the choice of priors and Markov chain Monte Carlo settings
☒	☐	For hierarchical and complex designs, identification of the appropriate level for tests and full reporting of outcomes
☐	☒	Estimates of effect sizes (e.g. Cohen's d, Pearson's r), indicating how they were calculated

Our web collection on statistics for biologists contains articles on many of the points above.

Software and code

Policy information about availability of computer code

Data collection	N4BiasCorrection: https://github.com/ANTsX/ANTs/releases/tag/v2.3.1; MASS: https://github.com/CBICA/MASS/releases/tag/1.1.1; MUSE: https://github.com/CBICA/MUSE/releases/tag/3.0.5 were used for MRI data preprocessing. Software for multi-view wealy-supervised clustering (Gene-SGAN model) is available as a published PyPI package GeneSGAN 0.0.2. Detailed requirement and instruction on implementation can be found at: https://pypi.org/project/GeneSGAN/. Custom code for GeneSGAN can be found at: https://github.com/zhijian-yang/GeneSGAN.
Data analysis	Codes for data analysis were based on online python packages, including scikit-learn 0.24.2; numpy 1.22.3; pandas 1.4.2; statsmodels 0.13.2; rpy2 3.5.1; nilearn 0.9.2, and the online R package: nnet 7.3-18. Online python package scikit-learn 0.24.2; SmileGAN 0.1.2; mvlearn 0.5.0 were used for model comparisons.

For manuscripts utilizing custom algorithms or software that are central to the research but not yet described in published literature, software must be made available to editors and reviewers. We strongly encourage code deposition in a community repository (e.g. GitHub). See the Nature Portfolio guidelines for submitting code & software for further information.

Data

Policy information about availability of data

All manuscripts must include a data availability statement. This statement should provide the following information, where applicable:

- Accession codes, unique identifiers, or web links for publicly available datasets
- A description of any restrictions on data availability
- For clinical datasets or third party data, please ensure that the statement adheres to our policy

The GWAS summary statistics generated in this study are provided in Supplementary Data files. Data used for this study were provided from several individual studies via data sharing agreements that did not include permission for us to further share the data. However, data from ADNI are available from the ADNI database (adni.loni.usc.edu) upon registration and compliance with the data usage agreement. Data from the UKBB are available upon request from the UKBB website (https://www.ukbiobank.ac.uk/). Data from the BLSA study are available upon request at https://www.blsa.nih.gov/how-apply. Data from the AIBL study are available upon request at https://aibl.org.au/. Data from the OASIS study are available upon request at https://www.oasis-brains.org/. Data requests for Biocard, Penn, and WRAP datasets should be directed to M.S.A, D.A.W, and S.C.J, respectively. Participant-level derived subtypes generated in this study will be provided within one month of receiving approval granted from respective studies.

Research involving human participants, their data, or biological material

Policy information about studies with human participants or human data. See also policy information about sex, gender (identity/presentation), and sexual orientation and race, ethnicity and racism.

Reporting on sex and gender	The sex distributions of participants from different studies are detailed in Table 1. There was no novel data collection. Information collection methods are defined directly by the ADNI, UKBB, BLSA, AIBL, BIOCARD, OASIS, PENN, and WRAP studies. We have examined and tested sex differences within each identified subtype of participants.
Reporting on race, ethnicity, or other socially relevant groupings	We did not use any socially constructed or socially relevant categorization variables in the manuscript. There was no novel data collection. Information collection methods are defined directly by the ADNI and UKBB studies. When testing SNP-subtype associations, the genetic principal components were included as covariates, adjusting for population structure and sample ancestry.
Population characteristics	Table 1 in the main manuscript provides the information of participants from different studies, including diagnosis, sex, and age. Genetic data is available for 1533 participants from the ADNI study and 27,325 participants from the UKBB study.
Recruitment	This study was a retrospective analysis. There was no novel participant recruitment.
Ethics oversight	The study was approved by the Institutional Review Board of the University of Pennsylvania.

Note that full information on the approval of the study protocol must also be provided in the manuscript.

Field-specific reporting

Please select the one below that is the best fit for your research. If you are not sure, read the appropriate sections before making your selection.

☒ Life sciences ☐ Behavioural & social sciences ☐ Ecological, evolutionary & environmental sciences

For a reference copy of the document with all sections, see nature.com/documents/nr-reporting-summary-flat.pdf

Life sciences study design

All studies must disclose on these points even when the disclosure is negative.

Sample size	Participants from ADNI, UKBB, BLSA, AIBL, BIOCARD, OASIS, PENN, and WRAP studies were included for the analysis. Detailed descriptions of studies, participants, and sample sizes can be found in Method 2 and Table 1 in the main manuscript. Since this study was a retrospective analysis, we did not predetermine the sample size, but used all available participants meeting the selection criteria as described in Method 6 and 7.
Data exclusions	Participants were included for semi-synthetic and real data experiments based on selection criteria and genetic quality check protocols detailed in Methods 5, 6, and 7.
Replication	In semi-synthetic experiments, we performed 20% leave-out cross-validation and evaluated the model's performance in identifying the simulated ground truth in test sets. In real-data experiments, we first tested the reproducibility of the identified subtypes through nested cross-validation and experiments with independent reference groups or patient groups. We further performed split-sampled experiments on the hypertensive population. We reported the replicability of imaging patterns associated with subtypes as well as the replication rate of identified subtype-genetic associations on the replication set.
Randomization	For 20% hold-out cross validation, we randomly left out 20% of the data each time we ran the Gene-SGAN model. For a 5-fold CV, we randomly divide the data into five folds and train the model using four folds of data. For the split-sampled experiments on the hypertensive population, we randomly half-divided data into a discovery set and a replication set.

Blinding	Since this study was a retrospective analysis, we did not collect any new data for a randomized controlled trial to test a clinical outcome. We performed our analyses on all available data meeting the criteria described in Methods 6 and 7, and blinding is not applicable to this study. Study methods used randomized sampling and cross-validation without direct user input.

Reporting for specific materials, systems and methods

We require information from authors about some types of materials, experimental systems and methods used in many studies. Here, indicate whether each material, system or method listed is relevant to your study. If you are not sure if a list item applies to your research, read the appropriate section before selecting a response.

Materials & experimental systems

n/a	Involved in the study	
☒	☐	Antibodies
☒	☐	Eukaryotic cell lines
☒	☐	Palaeontology and archaeology
☒	☐	Animals and other organisms
☐	☒	Clinical data
☒	☐	Dual use research of concern
☒	☐	Plants

Methods

n/a	Involved in the study	
☒	☐	ChIP-seq
☒	☐	Flow cytometry
☐	☒	MRI-based neuroimaging

Clinical data

Policy information about clinical studies

All manuscripts should comply with the ICMJE guidelines for publication of clinical research and a completed CONSORT checklist must be included with all submissions.

Clinical trial registration	Not applicable; not a prospective clinical trial.
Study protocol	Not applicable; this study was a retrospective analysis.
Data collection	There was no novel data collection. Data collection methods are defined directly by the ADNI, UKBB, BLSA, AIBL, BIOCARD, OASIS, PENN, and WRAP studies.
Outcomes	This study does not involve testing a clinical outcome.

Plants

Seed stocks	Not applicable
Novel plant genotypes	Not applicable
Authentication	Not applicable

Magnetic resonance imaging

Experimental design

Design type	Retrospective cohort study.
Design specifications	This is a retrospective study of data acquired by other research studies. All study data was available at the onset of this research study.
Behavioral performance measures	Clinical measures were obtained from the primary studies. There was no additional method to validate/verify clinical data.

Acquisition

Imaging type(s)	Structural MRI (T1, FLAIR)
Field strength	1.5 T and 3 T
Sequence & imaging parameters	MRI acquisition was controlled and performed by other studies; no new imaging was performed for this study. All studies have previously published methods for data acquisition.
Area of acquisition	A whole brain scan was used

Diffusion MRI ☐ Used ☒ Not used

Preprocessing

Preprocessing software	All image processing tools are publicly available as software packages that can be downloaded from public repositories.<br>N4BiasCorrection: https://github.com/ANTsX/ANTs/releases/tag/v2.3.1<br>MASS: https://github.com/CBICA/MASS/releases/tag/1.1.1<br>MUSE: https://github.com/CBICA/MUSE/releases/tag/3.0.5
Normalization	To generate the tissue density maps (RAVENS maps), we normalized the subject T1 scans to the MNI template and followed the procedure described in [1]. We used a non-linear deformation method [2] to normalize the images.<br>[1] Davatzikos, C., Genc, A., Xu, D. & Resnick, S. M. Voxel-based morphometry using the RAVENS maps: methods and validation using simulated longitudinal atrophy. NeuroImage 14, 1361-1369, doi:10.1006/nimg.2001.0937<br>[2] Ou, Y., Sotiras, A., Paragios, N., & Davatzikos, C. (2011). DRAMMS: Deformable registration via attribute matching and mutual-saliency weighting. Medical image analysis, 15(4), 622-639.
Normalization template	We used the MNI152 template, in the LPS orientation to normalize all subjects to a common space.
Noise and artifact removal	Multi-atlas ROI segmentation method is robust to noise and imaging artifacts because it's based on a consensus labeling approach, and also because it uses a deformable registration algorithm designed to reduce the negative impact of missing correspondences between images. Accordingly we did not need to apply further noise and artifact removal steps.
Volume censoring	Not applicable; Volume censoring is a preprocessing step specific for fMRI data and we don't use a technique equivalent to sMRI data.

Statistical modeling & inference

Model type and settings	Mass univariate voxel-wise group comparisons (CN vs each subtype) were performed via AFNI 3dttest using voxel-wise tissue density maps (RAVENs maps), adjusting for covariates including age, sex, and intracranial volume (ICV). To test associations between z2/3-PC and voxel-wise volumetric measures, we fitted mass univariate linear regression models with voxel-wise volumetric measures as dependent variables and each PC as an independent variable, adjusting for covariates including age, sex, ICV, and probabilities of subtypes.
Effect(s) tested	Voxel-wise volumetric measures in tissue density maps (RAVENS maps)[1]<br>[1] Davatzikos, C., Genc, A., Xu, D. & Resnick, S. M. Voxel-based morphometry using the RAVENS maps: methods and validation using simulated longitudinal atrophy. NeuroImage 14, 1361-1369, doi:10.1006/nimg.2001.0937

Specify type of analysis: ☐ Whole brain ☐ ROI-based ☒ Both

Anatomical location(s)	145 anatomical regions of interest (ROIs) were identified using a multi-atlas label fusion method[1]<br>[1]Doshi, J. et al. MUSE: MUlti-atlas region Segmentation utilizing Ensembles of registration algorithms and parameters, and locally optimal atlas selection. Neuroimage 127, 186-195, doi:10.1016/j.neuroimage.2015.11.073 (2016).
Statistic type for inference (See Eklund et al. 2016)	Voxel-wise group comparisons (CN vs each pattern) was performed via AFNI 3dttest using voxel-wise tissue density maps (RAVENs maps).
Correction	False discovery rate (FDR) correction for multiple comparisons with p-value threshold of 0.05 was applied.

Models & analysis

n/a	Involved in the study
☒	☐ Functional and/or effective connectivity
☒	☐ Graph analysis
☒	☐ Multivariate modeling or predictive analysis
